# Supplementary material for: Lc—ms-based untargeted metabolomics reveals potential mechanisms of histologic chronic inflammation promoting prostate hyperplasia
Source: PLoS One. 2024 Dec 23;19(12):e0314599. doi: 10.1371/journal.pone.0314599 (PMC11666064; doi:10.1371/journal.pone.0314599)
Supplement: Attachment — Submitted filename: Response to Reviewers.docx [file pone.0314599.s002.docx]

**Response to reviewers**

Dear editor and reviewers,

Thank you for offering us an opportunity to improve the quality of our submitted manuscript " Lc - ms-based untargeted metabolomics reveals potential mechanisms of histologic chronic inflammation promoting prostate hyperplasia" (ID: PONE-D-24-17979R1). I received constructive and insightful comments. In this revision, we have addressed all of these suggestions. We hope the revised manuscript has now met the publication standard of your journal. We highlighted all the revisions in red colour. Below are our responses to the questions posed by the reviewers.

**Overall comment 1**

**The authors should provide the full forms and significance of all abbreviations (e.g., PV) to enhance comprehension for general readers.**

Response: Thank you for your suggestions. We have carefully and meticulously refined the full names of all abbreviations in this paper. We have red-flagged the changes in the article.

**Comment 2**

**Were there any challenges related to ion suppression or enhancement during the LC-MS analysis, and what strategies were employed to address these effects?**

Response: Thank you very much for your professional question. Ion suppression or enhancement is a manifestation of matrix effect, which does exist during LC-MS analysis. The matrix effect in LC-MS is generally thought to occur because non-volatile components of the matrix compete with the substance to be measured, during ionization on the surface of the droplet, affecting the ionization efficiency at the electrospray interface. These non-volatile matrix components draw the droplets together, preventing them from cleaving into smaller microdroplets. The interfering substances that produce matrix effects fall into two categories. One group is called “endogenous substances”: substances that originate from the analyte itself and remain in the final extract, including salts, strongly polar compounds, surfactants, and lipids, amines, peptides, etc., that are structurally similar to the target compound. The other category is called “exogenous substances”: they do not come from the matrix itself, but from the external environment during the establishment of the method, including plastic and polymer residues, ion-pairing reagents, organic acids, buffer solutions, etc. In this experiment, I mainly take the substances from the matrix itself and retain them in the final extraction solution. In this experiment, I took four main programs to reduce the matrix effect. First, we adopted the better extraction scheme of liquid-liquid extraction for tissue metabolites as tested by want et al. The principle is that nonpolar compounds are more soluble in organic solvents than in water. On the contrary, ionic or polar compounds are more soluble in aqueous solutions than in organic solvents. Ultimately, the polar and nonpolar compounds will be extracted as much as possible. Second, after preexperimentation, we applied a variety of columns (HSS T3, BEH C8, BEH C18, CSH Phenyl-Hexyl) with different chromatographic separation conditions. It was found that the application of the columns and chromatographic conditions in this study had minimal matrix effects. Third, we used leucine enkephalin for mass calibration to reduce mass bias. Fourth, Progenesis QI (Waters, Nonlinear Dynamics, Newcastle, UK) is a better metabolomics data processing software, which has the functions of base; line filtering, peak alignment and deconvolution to reduce the experimental result error caused by matrix effect. Due to the fixed equipment, the replacement of ion source was not carried out in this experiment, and it was not possible to assess the extent of the matrix effect of most compounds on the ion source. These are the measures I took to reduce the matrix effect.

**Comment 3**

**Can the authors elaborate on the limitations encountered during the sample preparation process and how they were addressed to ensure the reliability of the LC-MS results?**

Response: Thanks again for the question from the expert, there are indeed many limitations in the sample preparation process. The main ones are following items. First, sample contamination and sample stability. After the patient underwent prostate enlargement surgery, we collected the prostate tissue in sterile, enzyme-free cryopreservation tubes within 30 minutes and stored it in a -80°C refrigerator in the operating room. The vast majority of metabolites will not be degraded and transformed under ultra-low temperature conditions. Second, loss during sample processing. During sample processing, loss of target metabolites may occur due to the different extraction protocols for metabolites and the time required. Therefore, we performed pre-experimentation beforehand to choose a better extraction protocol and operated on dry ice throughout the process to reduce the loss of metabolites. Third, matrix effect. We ensured that all reagents and materials were of high quality and met the requirements for LC-MS analysis. Plastic devices that could interfere, such as bottles, funnels, beakers, etc., were avoided. Likewise the measures taken in the second expert question. Fourth, strict quality control. In the pre-treatment, the original samples were randomly disrupted between all the different groups to avoid batch effects between the comparison groups. For quality control (QC) and to improve the stability of the system, 10 μL of supernatant was transferred from each sample and mixed as QC, and 18.2 MΩ.cm pure water was used as a blank control group, and the QC samples and the blank control group would be injected at regular intervals (every 5 samples) to minimize the sample residues and to monitor the experimental stability. A blank solvent (Blank) was used to balance the instrument status and clean up the column residual material during the assay to avoid sample residue and contamination buildup problems. Preventive maintenance is achieved by pre-collecting QC mixed matrix samples to prevent situations such as unstable data.

[Author Name]: JiaLe Li

[Affiliation]: Clinical Medical College, North China University of Science and Technology

[Mailing Address]: Tangshan, Hebei, China, 063210

[Phone Number]: +86 18352185307

[Email]: 1654989167@qq.com
